# Young black women’s desired pregnancy and birthing support during coronavirus disease 2019 pandemic

**DOI:** 10.1016/j.ssmqr.2023.100333

**Published:** 2023-09-09

**Authors:** Ashley V. Hill, Phoebe Balascio, Mikaela Moore, Fahmida Hossain, Megana Dwarkananth, Natacha M. De Genna

**Affiliations:** a Department of Epidemiology, University of Pittsburgh School of Public Health, Pittsburgh, PA, USA; b Department of Psychiatry, University of Pittsburgh School of Medicine, Pittsburgh, PA, USA; c Department of Pediatrics, University of Pittsburgh School of Medicine, Pittsburgh, PA, USA

## Abstract

**Objective:**

To document pregnancy and birthing experiences of young, Black pregnant women in one geographic area to make recommendations for improving young Black women’s pregnancy and birthing experiences.

**Methods:**

Participants were recruited through The YoungMoms Study (R01 DA04640101A1) in Pittsburgh, Pennsylvania, and included Black or biracial participants ages 16–23 (n = 25). Individual interviews were conducted from March 2022–July 2022 to assess pre-, peri-, and post-natal healthcare system encounters; experiences of structural and obstetric racism and discrimination in healthcare settings while obtaining prenatal care; attitudes around healthcare systems and medical professionals; effects of COVID-19 pandemic on participants lives and the impact of enacted healthcare policies in their perinatal experience; substance use changes during pregnancy; and coping mechanisms for stress. NVivo 13 was used to code transcripts, then major themes and subthemes were identified using thematic content analysis and based on grounded theory.

**Results:**

Twenty-five interviews were conducted, and four themes emerged from participant experiences of racial discrimination in healthcare settings; (1) awareness of historical racism that influences perinatal care; (2) clinical providers assume participant substance use and enact reproductive coercion; (3) clinical providers question validity of Black women’s birthing complaint; and (4) Young Black pregnant women know and will express what they desire in their perinatal experience if asked.

**Conclusions:**

Young Black pregnant women encounter structural racism and intersectional bias from healthcare providers. By centering the perspectives and experiences of this overlooked population, public health researchers and clinical providers can utilize anti-racist frameworks to create more equitable, just practices in reproductive healthcare.

## 1. Introduction

The coronavirus disease pandemic (COVID-19) solidified the impact of structural racism and discrimination on Black maternal health (Bailey et al., 2017; Balascio et al., 2023; Johnson-Agbakwu et al., 2022). Black women of all ages experience a maternal mortality rate more than double that of the national average (55.3 versus 23.8 per 100,000) in the United States (US) (Hoyert, 2022/02) and are at increased risk of pre-eclampsia (Boakye et al., 2021), preterm birth (Braveman et al., 2021), and low birthweight (Ratnasiri et al., 2018). These outcomes have been attributed to anti-Black obstetric and medical racism (Davis, 2019; Scott & Davis, 2021), linked to historical experiences of Black women in the US (Owens & Fett, 2019; Prather et al., 2016, Prather et al., 2018). Perinatal studies have described links between racialized experiences in the US and adverse pregnancy, neonatal and maternal outcomes (Arora et al., 2020), prompting the urgency to address racism at multiple levels of healthcare (Adkins-Jackson et al., 2022).

Additionally, recent data suggests younger pregnant women aged 11–19 had a trending increase in risk for postpartum hemorrhage, hypertensive disorders of pregnancy and severe maternal morbidity from 2000 to 2018 (Staniczenko et al., 2022). These demonstrated risks of adverse pregnancy outcomes in both younger and Black populations suggests that Black young birthing people may have unique and multiple challenges in their pregnancies. Therefore, elucidating factors contributing to adverse outcomes in younger minority women, in addition to the societal infrastructures and compounding social factors (e.g. racism, sexism, experiences with violence) (Bowleg, 2012) that influence those outcomes is necessary to reduce these stark disparities.

Studies examining the unique challenges facing young (age <21), Black, pregnant women in the US are limited, but necessary to improve outcomes. Qualitative examinations of intersectional experiences are known to be helpful in providing robust descriptions of marginalized experiences for translational interventions (Brand et al., 2014; Kane et al., 2014). Therefore, the purpose of this work is to document pregnancy and birthing experiences of young, Black pregnant women during the COVID-19 pandemic in one geographic area to make recommendations for improving pregnancy and birthing experiences.

## 2. Methods

### 2.1. Study design

Participants were recruited through The YoungMoms Study (R01 DA04640101A1), a longitudinal cohort study designed to examine perinatal cannabis and tobacco co-use in young people and possible associated outcomes for infants. YoungMoms uses a mixed-method, multiple reporter (e.g., anonymous tablet surveys, interviews, medical records, biological testing) approach to collect quantitative and qualitative data to assess prenatal cannabis exposure and co-use with tobacco in pregnant adolescents and young adults. Young women recruited into the parent study are: (1) 13–21 years old; (2) confirmed pregnant; (3) less than 14 weeks gestation; (4) present to one of the sites for an initial prenatal visit; (5) are able to read and speak English; (6) live close enough to the study site to return for follow-up visits postpartum; and (7) could provide informed consent. For this qualitative analysis with grounded theory methods (Jørgensen, 2001; Khan, 2014), we recruited a subset of Black and biracial participants who were not enrolled in the YoungMoms longitudinal study because they were >14 weeks gestation at baseline. The PI conducted this study during the COVID-19 pandemic, and included several questions about experiences and impacts on pregnancy and birthing. Pandemic related restrictions widely affected all birthing people and included restrictions to support persons in hospital settings, rapidly changing and unclear guidelines, and increased stress due to restrictions (Altman et al., 2021; Davis-Floyd et al., 2020). These systems changes may have uniquely impacted younger Black birthing people and questions were included in the interview guide to ascertain these impacts. This subset was approached to participate in individual interviews investigating their pregnancy and birthing experiences during the COVID-19 pandemic, including discrimination and instances of structural and obstetric racism (Davis, 2019; Scott & Davis, 2021), pandemic related stress, and changes in substance use during COVID-19. All research activities were reviewed and approved by the institutional review board.

### 2.2. Recruitment and context

Obstetric patients ages 13–21 who lived in Pittsburgh, Pennsylvania (PA) and were recruited for the YoungMoms study from a regional medical clinic and teaching hospital that provides services to patients with public benefits. To reduce participant burden, Black and biracial patients who were not enrolled into the longitudinal study were contacted for remote qualitative interviews by telephone between January–March 2022. Of the 32 people contacted about the study, 25 agreed to participate in the interviews, 2 refused, and 5 were never reached. A detailed description of the research study was provided to those who agreed to participate, confidentiality of data was discussed, and consent for participation and recording of interviews was obtained prior to proceeding. Upon study completion, participants were offered a $50 gift card compensation. Recruitment continued until thematic saturation was reached, as determined by research group consensus. Interview data were collected between March 2022–July 2022.

### 2.3. Interview guide

The semi-structured interview guide was developed by two senior investigators. Themes of interest included: pre-, peri-, and post-natal healthcare system encounters; experiences of structural and obstetric racism and discrimination in healthcare settings while obtaining prenatal care (Davis, 2019); attitudes around healthcare systems and medical professionals; effects of COVID-19 pandemic on participants lives and the impact of enacted healthcare policies in their perinatal experience; substance use changes during pregnancy; and coping mechanisms for stress (Appendix 1).

### 2.4. Data collection

One clinician and one social sciences researcher with qualitative research backgrounds conducted one-to-one independent interviews. Semi-structured, individual interviews lasted 45–60 min and were conducted via Zoom. All participants provided verbal informed consent; after receiving consent, interviewers recorded the Zoom meeting with the built-in “record meeting” feature. Upon interview completion, members of the study team manually transcribed audio files using the Zoom meeting built-in “transcription” feature as a guide. Two interviews were professionally transcribed due to research team capacity. All files were de-identified.

### 2.5. Data analysis

NVivo 13 (released March 2020) was used for qualitative data analysis. Major themes and subthemes were identified using grounded theory and thematic content analysis (Keddy et al., 1996; Noble & Mitchell, 2016). To develop a preliminary codebook, two teams of two investigators coded five initial interviews separately, then codebooks were compared for agreement and finalized through consensus. As themes emerged from subsequent interviews, iterative developments to the codebook were completed with group consensus until thematic saturation was reached.

### 2.6. Reflexivity

The research team included diverse academic, clinical, educational, racial/ethnic, sexual orientation, and parenting backgrounds. Two study members conducted all interviews; both are racially marginalized women although neither identified as Black. Coding was completed by four team members, three of whom are racially marginalized women.

## 3. Results

### 3.1. Participant demographics

Participants (n = 25) were ages 16–23 years old when they were interviewed with the majority (88.0%) identifying as non-Hispanic Black (Table 1). Forty percent of participants had a previous pregnancy, most were not employed, and about a quarter (24.0%) were still in high school when they completed the electronic survey. Most participants (88.0%) received Medicaid benefits. All participants gave birth prior to their interview.

### 3.2. Thematic results

Participant experiences of racial discrimination and desires in healthcare settings emerged, and concepts were categorized into four groups (Table 2).

#### Theme 1: Participant awareness of historical racism and discrimination in healthcare and influencers of perinatal care.

In contextualizing their experiences of medical racism, participants described how slavery and white supremacy in medicine permeate the discipline. One participant shared how histories of racist medical research continue to influence racial discrimination in current medical education and healthcare:

“I think one of the reasons that healthcare providers have issues with women of color now […] there was that idea for a long time, that Black people could tolerate pain better than white people. And I feel like that’s kind of something that leads into the healthcare situation now, with people not listening to black women when they say they’re in pain, or this is what they want to do.”

– Participant 5059

Participants attributed these historical experiences to contemporary racial injustices in reproductive health outcomes. Importantly, knowledge of how Black women are treated in medical settings also coincided with fear and anxiety of healthcare practitioners.

“I’ve seen a lot of videos where females of color, or just colored people just would pass [away] because some doctors, not all of them, will just be careless. I do feel as though that is a problem. So yes, I was very scared. I was.“

- Participant 5189

Another participant described:

“I was reading an article that women of color, when they go through pregnancy […] die. Some […] medical providers don’t take their problem serious[ly]. And [that] adds up to death. It just scared me. I don’t want to […] be pregnant ever again […] I feel like voicing some of my concerns they weren’t really concerned or it’s just like, “Oh that’s normal during pregnancy.“

- Participant 5209

Young Black women are knowledgeable about how the medical field, and particularly reproductive medical care, is rooted in anti-Black practices and continues to produce stark inequities in perinatal and maternal outcomes. With this understanding, patients report feelings of fear and anxiety of not being believed or taken seriously in medical settings.

#### Theme 2: Clinician bias in assumptions of substance use and reproductive coercion from clinical providers.

Patients expressed experiences of discrimination from medical providers throughout their reproductive health care. One participant described judgment, discrimination, and reproductive injustice from a medical provider when seeking contraceptive care:

“When I was 18, I went to get an [intrauterine device] IUD. Just because I had really bad cramps with my cycle and I just wanted to be safe because I did not want to have a baby at that time in my life and accidents do happen. […] The doctor that I was trying to get my IUD was very unhappy with the fact that I was 18 and she expressed how she feels that I should be focusing on school and sex should be the last thing on my mind and I’m too young […] So she did not do the implant […].“

- Participant 5294

This study particispant further described how this negative encounter impacted their access to reproductive care in a timely way:

“I had to switch doctors because she was not happy with my decision to get an IUD. And that led me to have a more uncomfortable visit with a male doctor because I’m not comfortable with male doctors around male doctors and a male doctor had to put the implant which I had a huge panic attack, and that was a whole fiasco. Just because she was unhappy with me getting an IUD at 18. Even though as a doctor it’s not her- this is not her choice to tell me what she thinks I should do with my body. She- if I came to get IUD, that’s what she should have done instead of showing me and telling me how unhappy she was with me.“

- Participant 5294

In this participant’s experience, this challenging encounter with a medical provider led to a further traumatizing experience with contraceptive care. This denial of access and autonomy required them to be overwhelmingly persistent in the face of an already challenging landscape of reproductive justice. Contraceptive care and counseling may be heavily influenced by provider biases. Participants shared that physicians pressured them to use birth control once their baby was born, sometimes under the guise of caring for their wellbeing:

“They [physicians] were just pressuring me to get on birth control stuff […] they don’t want me to have any more babies, because I’m young […] I didn’t want to get on birth control. I kept on telling them that […] I wasn’t doing anything, and I’m not going to get pregnant again anytime soon, but they’re still just pressuring me. […] That OK you’ve already got two babies.“

- Participant 5043

Feeling forced or pressured in contraceptive care by doctors was a commonly discussed challenge by participants:

“Because I was young, they automatically assumed I didn’t want to have the baby. […] I didn’t like the fact that they just put a number over my head and automatically felt like they knew me.”

– Participant 5101

Additionally, clinicians were described as trying to influence participants to release custody of their child:

“They’re giving you options, but they were really keen on me giving up my baby. That was their main thing […] they kept talking about adoption. But when I told them I wanted the kid but they were like, “Are you sure?” They didn’t know if I was sure about keeping my baby.“

- Participant 5101

In addition to reproductive coercion reported by participants, several shared facing stereotypes of substance use:

“They [doctors] kept making it seem like I was doing substances and I wasn’t, so that was annoying me. Also they kept thinking the reason why […] I wasn’t gaining weight because I’m doing substance. So they kept asking me about weed and all that and I was like “I have to switch hospitals.“

- Participant 5101

Participants experiences of being judged or screened for substance use were overwhelming, such that one changed care teams because of this behavior from clinicians.

#### Theme 3: Clinical providers question validity of Black birthing complaints.

Patients often described feeling unheard and not believed when expressing issues to their providers. These encounters often induced fear for their or their baby’s health and safety:

“It was just the whole situation, the birth situation. […] I was scared through the whole experience, I was scared the whole time. And I just feel like I was being treated like I wasn’t there. They knew I was scared, and they were just doing a lot of things like talking to themselves, not letting me know what they’re doing before they’re doing it.“

- Participant 5165

Many patients described the need to self-censor or act in ways that would be more acceptable to medical providers, with the goal of ensuring their needs were met:

“I feel like, with people of darker skin color, we have a hard time expressing what we want because then we come off as greedy or asking for too much […] We can’t say it outright […] I’ve never been able to go into a doctor’s office and be like, “Hey, this is how I feel, this is what I think’s going on.” No, I’m quiet when I go into the doctors, I’m questioning not only them but myself.“

- Participant 5059

Others described how being unheard or not given information created other emotional responses:

“[I was] scared and just aggravated and angry, and traumatized, my body was shaking the whole entire time, and I’m saying things out of anger. I don’t mean to be aggressive, but that was the only way for them to hear me. It took me a while to get stitched up, […] and I feel like they were getting annoyed with me. It was just a whole weird situation. I knew if it was a white woman or something, they probably would have heard her. It would probably have been a whole different situation.“

- Participant 5165

Young Black birthers identified specific instances of inequitable pregnancy care and clearly described the emotional and psychological stress and distress from these experiences.

#### Theme 4: Young Black birthers know and will express what they desire in their perinatal experience if asked.

When asked for suggestions to improve encounters with the healthcare system and reduce incidents of racial discrimination, participants often responded with an appreciation for being asked, as typical encounters in clinical care lack opportunities and invitations for patient input. Participants shared a desire to have increased diversity among providers:

“I honestly didn’t have any type of doctor that was like around my color, or just honestly, just any other type of like color. They were just all white.“

- Participant 5189

Another described:

“I wish they didn’t look at me so much because of my age. I feel like they looked at me so much […] as hard as it is for you to think that I’m this young pregnant, it’s hard for me too. They didn’t think about the fact that staring gets annoying.

– Participant 5101

Participants would like to see clinicians receive additional education in bedside manner and histories of medical racism:

“I would love for people [doctors] to look back [into history] and really think on everything that’s happened so that they can actually, maybe just change and realize these woman [Black women] been through enough.“

- Participant 5071

To improve care provision, participants describe a desire for clinicians to learn historical perspectives, to be culturally representative, and to genuinely respond to patient concerns.

## 4. Discussion

This qualitative study examined pregnancy and birthing experiences of Black pregnant young women between the ages of 16–21 in Pittsburgh, Pennsylvania. Primary themes emerged related to (1) patient awareness of histories of racism and influences on healthcare of Black women, (2) clinician assumptions of substance use and reproductive coercion, (3) clinicians questioning validity of patient complaints, and (4) patient desires to be asked by clinicians and perinatal support persons what Black women want from their pregnancy and birthing experience. This study elucidated how intersectional experiences (e.g., race, gender, and age) (Essed, 2001) impact encounters within the healthcare system and may result in adverse reproductive outcomes. Measuring structural racism, racial discrimination, and their impacts to population, community, and individual health is imperative to dismantling these systems of power (Adkins-Jackson et al., 2022; Paradies et al., 2014).

Providers must take on the responsibility to reflect on implicit and explicit manifestations of racism and discrimination in their practice. Suggestions to move towards this integration have been proposed. For instance, Hardeman and colleagues propose four “Relationship-centered care principles” to balance inherent provider-patient power dynamics and improve pregnancy outcomes of Black women: (1) dimensions of personhood and roles; (2) affect and emotion are important components; (3) context of reciprocal influence; (4) health care has a moral foundation (Hardeman et al., 2020). Rooted in critical race theory, Hardeman et al. writes that clinicians must practice racial consciousness, outwardly demonstrate empathy, promote shared decision-making, and authentically integrate racial justice as a core tenet of medicine. Additionally, other researchers have suggested approaches to improving quality of care for Black adults in the United States rooted in anti-racist approaches (Headen et al., 2022; Legha et al., 2020). These needs are echoed by our participants, who shared the need for complex strategies to navigate racism in obstetric care (addressing clinician bias and algorithms for service referral), desires for practitioners to understand medical racism as a historical context and lived experience, and wishes for clinicians to prioritize patient autonomy.

Events during the pandemic sparked a national conversation across the US about the impacts of racism on health, with medical professionals highlighting the needs to address racism and improve racial equity in the field (Mendez et al., 2021). Thus, a crossroads presents itself in obstetric and gynecologic medical care. Given the disparities in Black maternal and infant outcomes, the pledges of healthcare providers to better serve Black communities, the existing health and social inequities illuminated by COVID-19, and a wealth of Black-led scholarship towards anti-racist health research, it is necessary to capitalize on the present moment to understand how Black birthing people continue to experience racial discrimination in their medical care during pregnancy (Galea & Abdalla, 2020; Njoroge et al.). By integrating approaches such as the “Relationship-centered care principles” (Hardeman et al., 2020), researchers and clinicians can advance racial health equity in pregnancy care, particularly for young Black mothers, and reduce patients’ experiences of racial discrimination in medical settings.

### 4.1. Strengths and limitations

Participants were recruited from a women’s clinic and clinician training center that primarily serves socioeconomically marginalized birthers from Western PA in the US. Interviews were facilitated by non-Black women of color in academia; given that all participants were young Black women, respondents may have influenced by social desirability bias.

This qualitative study is a timely reflection of how young, Black pregnant women experienced discrimination and structural racism in reproductive and maternal healthcare. Participants were able to express negative opinions and experiences, along with positive encounters and desired supports. Grievances expressed in the study were uplifted using the participant’s own words, particularly among a population of focus that has historically been excluded from research studies of pregnancy.

## 5. Conclusion

Young Black pregnant women described themes of structural racism and discrimination from healthcare providers during their pregnancy experiences. Future research should identify and implement strategies to eliminate obstetric racism and determine effective protocols to advance racial justice at all levels of care.

## Supplementary Material

1

## Figures and Tables

**Table 1:** Characteristics of the YoungMoms qualitative sample (n = 25).

Participant Characteristics	n (%)
Age (years)	
<18	3 (12.0)
≥18	22 (88.0)
Race	
Biracial	3 (12.0)
Black	22 (88.0)
Ethnicity	
Hispanic/Latino	1 (4.0)
Not Hispanic/Latino	24 (96.0)
Gender Identity	
Girl	25 (100.0)
Pregnancy History	
First pregnancy	16 (60.0)
First birth	9 (37.5)
Enrolled in school	6 (24.0)
Employment	
Part-time	1 (4.0)
Full-time	5 (20.0)
Not employed	19 (76.0)
Insurance	
Medicaid	22 (88.0)
Private	3 (12.0)
Living circumstances	
With parents	10 (40.0)
Alone with child/ren	9 (36.0)
With co-parent/father of the baby	4 (16.0)
With another relative	1 (4.0)
Unspecified	1 (4.0)

**Table 2:** Emerging themes and exemplary quotes related to racial discrimination in healthcare during pregnancy.

Theme	Exemplary quote
Participant awareness of historical racism and discrimination in healthcare and influencers of perinatal care.	We [Black Americans] came from being slaves at one point in our lives and being raped by slave masters, […] **You have to think about what we went through, in the past of being stolen, of being able to say even sexually, even being able to say yes or no.**
Clinician bias in assumptions of substance use and reproductive coercion from clinical providers.	**My doctor kept kind of trying to force me on birth control too.** So, I feel like it was […] a caring type of thing, but I also seen them looking down on me because I was a young mom.
Clinical providers question validity of Black birthing complaints.	They’ll [doctors] be like, “Have you been experiencing these symptoms?” I don’t know, have I? Am I maybe making this up? We [Black women] start to get into our heads about whether or not what we’re feeling is genuinely what we’re feeling […] **I feel like if we go to doctors, we’re more likely to be gaslit than we are to be attended to**.
Young Black birthers know and will express what they desire in their perinatal experience if asked	Because all these women regardless, what color they are, every woman that becomes pregnant and, and has to go to a hospital and give birth, even the ones that get pregnant and don’t have a baby. Maybe miscarry, you’re getting an abortion or whatever have give birth to a stillborn. **They all deserve dignity, respect, and to be treated like human beings and not just lab rats.**
