# Supplementary material for: Young black women’s desired pregnancy and birthing support during coronavirus disease 2019 pandemic
Source: SSM Qual Res Health. Author manuscript; Available in PMC 2023 Dec 15. (PMC10722556; doi:10.1016/j.ssmqr.2023.100333)
Supplement: 1 [file NIHMS1950284-supplement-1.docx]

**Impacts of COVID-19 and Structural Racism on Pregnancy Interview Guide**

Thank you for agreeing to participate in this interview today and taking the time to speak with us.

**Purpose:** We ask for your help in understanding the impact of racism and discrimination during pregnancy, especially during the pandemic. The questions we ask are to better understand your pregnancy experiences, medical visits, and birthing experiences. Your answers will help us create some strategies that can respond to your experiences and work on making improvements in care. What you say is valuable to us, and we want to learn from you.

**Confidentiality:**

There are no right or wrong answers, so please feel free to answer honestly. We just want to know what you think and your opinions. You won’t hurt our feelings or affect us in any way.

Please, don’t worry about anything we will discuss during this interview. No personal information will be connected to anything you say here today, and everything will be kept confidential. Nothing will be shared with your health care providers.

We will be recording this interview, and only project staff will have access to these recordings, if that’s OK with you?

We really appreciate you talking to us today and know that we’re asking you to talk about some sensitive topics. Please know that we will not judge you for anything you say here and just want to know how we and other people can support young pregnant women.

Do we have your consent to start the interview and record?

[start recording]

An Ice Breaking Question:

How are you doing?

Before we begin, do you have any questions?

General questions about the healthcare system

**What were your experiences like with the healthcare system during your pregnancy, labor, delivery and afterwards?**

Probes:

i. What good experiences did you have?

ii. What bad experience did you have?

iii. Are there ways the care could have been better?

iv. Who helped you during your pregnancy? Was there a support person at your appointments/visits (who was this person/people, e.g., partner, parent, foster parent, sister, doula, advocate)?

Ask Supporting Questions: Tell me more about it. What did that look like? Walk me through your visits! Can you share more details, specifics?)

COVID pandemic

For the next few questions, I want to ask your experiences during the pandemic.

**How has it been for you and your family since the beginning of the pandemic in March 2020?**

Probes:

i. Were you or someone in your family at greater risk of infection because of their job situation?

ii. Did the pandemic create financial hardships (food insecurity, loss/change of housing, role and usefulness of COVID-related government assistance)

iii. In what ways did the pandemic affect your childcare situation (how)?

iv. What helped you to cope? Anything in particular? Any support system?

v. How have the Covid vaccines influenced you or your family?

Ask Supporting Questions: Tell me more about it. What did that look like? Can you share more details, specifics?)

Now we are going to talk about substance use, and I want to remind you that we are not going to share this recording or anything you say with your healthcare providers or anybody outside the study. You are the expert, you have gone through the experience, and we want to learn from you. We want to figure out how to better help people during pregnancy, especially given Covid and all those challenges.

Substance use and coping during the pandemic

**Many people felt stressed and found various ways to cope with their stress and anxiety during the pandemic. What were things that you did to cope during the pandemic? Would you say that your habits changed or pretty much stayed the same or did they change?**

Probes:

i. Changed – how? Why do you think that happened?

ii. Stayed the same – why do you think some people changed and you did not?

[if they don’t mention substance use, probe for it by asking – what about smoking or drinking?]

Obstetric Racism

**1. Can you recall a time when you were treated with less courtesy/respect by a doctor, nurse or anyone else in the clinic, that you felt was because of your race? [did you experience anything you thought was directly racial or biased during your care?]**

[Do you feel this treatment may have been because of your age?]

[Did you feel like your doctor was not listening to you or taking you seriously]

**2. Can you share any positive experience with a doctor, nurse, or anyone else at the clinic?**

[What specifically did they do that made you feel good/supported/listened to?]

**3. What do you wish all healthcare providers knew about pregnant Black/Biracial women?**

**4. If you can make any changes in pregnancy care what would they be, what would those changes look like?**

**5. Are there other ways you felt unheard, unseen, judged, and overlooked or felt lesser during your pregnancy and care? (transportation/ neighbors/ making appointments/ community.)**

Closing

**1. Is there anything else you wish I had asked about that I didn’t?**

**2. Do you have any questions for me?**

Thank you for taking the time to share your experiences today. We will be using this

information to develop survey questions about the pandemic and discrimination in health care. At some point, we hope to share the measures we develop based on these discussions and get some feedback from you to make sure we accurately captured your thoughts. Any last thoughts or questions before I stop the recording?
